# Case Report: Successful weaning after 113 days of VV-ECMO in a pediatric patient with severe ARDS following Stevens–Johnson syndrome

**DOI:** 10.3389/fped.2025.1691727

**Published:** 2025-12-12

**Authors:** Korbinian Beil, Matthias Hermann, Nikolaus Haas, Robert Dalla-Pozza, Sebastian Michel, Andre Jakob, Marcus Fischer, Joseph Pattathu

**Affiliations:** 1 Department of Pediatric Cardiology and Intensive Care, LMU Munich University Hospital, Munich, Germany; 2 Department of Thoracic Transplantation in Children, LMU Munich University Hospital, Munich, Germany; 3 Department of Surgery of Congenital Heart Defects, LMU Munich University Hospital, Munich, Germany

**Keywords:** pediatric acute respiratory distress, extracorporeal membrane oxygenation, Stevens–Johnson syndrome, prolonged ECMO weaning, case report

## Abstract

Veno-venous extracorporeal membrane oxygenation (VV-ECMO) is an established rescue therapy for severe pediatric acute respiratory distress syndrome (ARDS), but prolonged support is rarely reported. We describe the case of a previously healthy 6-year-old boy who developed Stevens–Johnson syndrome (SJS), complicated by progressive respiratory failure and severe ARDS. Despite maximal ventilation, oxygenation remained insufficient, and VV-ECMO was initiated on day 11 of illness. Cannulation was performed via jugular and femoral access, followed by lung-protective ventilation, repeated surfactant administration, corticosteroid therapy according to the Meduri protocol, and angiotensin-converting enzyme inhibitor therapy. ECMO support was complicated by pulmonary fibrosis, cholestatic liver dysfunction with secondary hemochromatosis, and prolonged sedation-associated delirium with subsequent critical illness polyneuropathy. The first attempt to discontinue ECMO after 90 days failed due to presumed pulmonary embolism, requiring recannulation. Ultimately, successful weaning was achieved after 113 days of VV-ECMO. The patient was transferred to a specialized pulmonary and neurological rehabilitation center and discharged home after 6 months, still dependent on a tracheostomy cannula. At the 18-month follow-up, he required only nocturnal mechanical ventilation through the tracheostomy, was attending school, and led an otherwise normal life. A trial removal of the cannula with closure of the stoma is scheduled for spring 2026. This case illustrates that prolonged VV-ECMO can allow lung recovery in pediatric patients with ARDS secondary to SJS, despite complications. Careful multidisciplinary management and preserved neurological function were key factors supporting long-term survival.

## Introduction

Pediatric acute respiratory distress syndrome (ARDS) is rare (incidence ∼5.5/100,000 in Germany) but carries a mortality of about 20%, almost always requiring intensive care (1, 2). Most cases result from pneumonia, systemic infections, or aspiration; rarer causes include trauma, near drowning, or medications (3).

Stevens–Johnson syndrome (SJS) is an uncommon, drug-induced disorder characterized by epidermal and mucosal detachment (4, 5). Involvement of the bronchial mucosa is unusual but can trigger severe ARDS (6, 7). In cases where mechanical ventilation fails to maintain gas exchange, veno-venous extracorporeal membrane oxygenation (VV-ECMO) may be required to minimize ventilator-associated damage until lung recovery occurs.

While most pediatric ECMO runs last days to 2 weeks, prolonged support beyond 28 days has historically been associated with poor outcomes. In 2012, Gupta et al. reported that among 951 children treated with ECMO, only 22 required support for ≥28 days, with survival achieved in just a small fraction of those cases (8).

Nonetheless, isolated case reports have demonstrated successful recovery after extended ECMO runs, including pediatric courses of 30–86 days, suggesting that lung healing can still occur after prolonged periods of apparent stagnation (9, 10). Reports of pediatric SJS complicated by severe ARDS requiring ECMO are exceedingly rare, making our case particularly noteworthy. In addition to the medical challenges, such extreme ECMO durations inevitably raise ethical considerations regarding proportionality of care, resource allocation, and the balance between potential recovery and treatment burden. We present this case to add to the limited body of literature describing prolonged ECMO in children and highlight both the clinical and ethical aspects of long-term extracorporeal support.

## Case report

### Initial presentation

A previously healthy 6-year-old Caucasian boy with no relevant past medical or familial history presented with fever up to 40 °C, conjunctivitis, and rash. There was no known family history of allergic or drug-related reactions. Initial antibiotic therapy with cefpodoxime was started for suspected streptococcal infection. However, as his condition worsened, he was treated for suspected Kawasaki syndrome with aspirin and intravenous immunoglobulins. Shortly thereafter, he developed severe epidermolysis. Skin biopsy confirmed Stevens–Johnson syndrome. Ciclosporin A and corticosteroids were initiated, but respiratory failure developed within 3 days. No definitive drug trigger could be identified despite extensive examination of the medication history. By day 7, he required intubation, and on day 11, he was transferred to our ECMO center.

### Clinical findings

**Figure 1:**
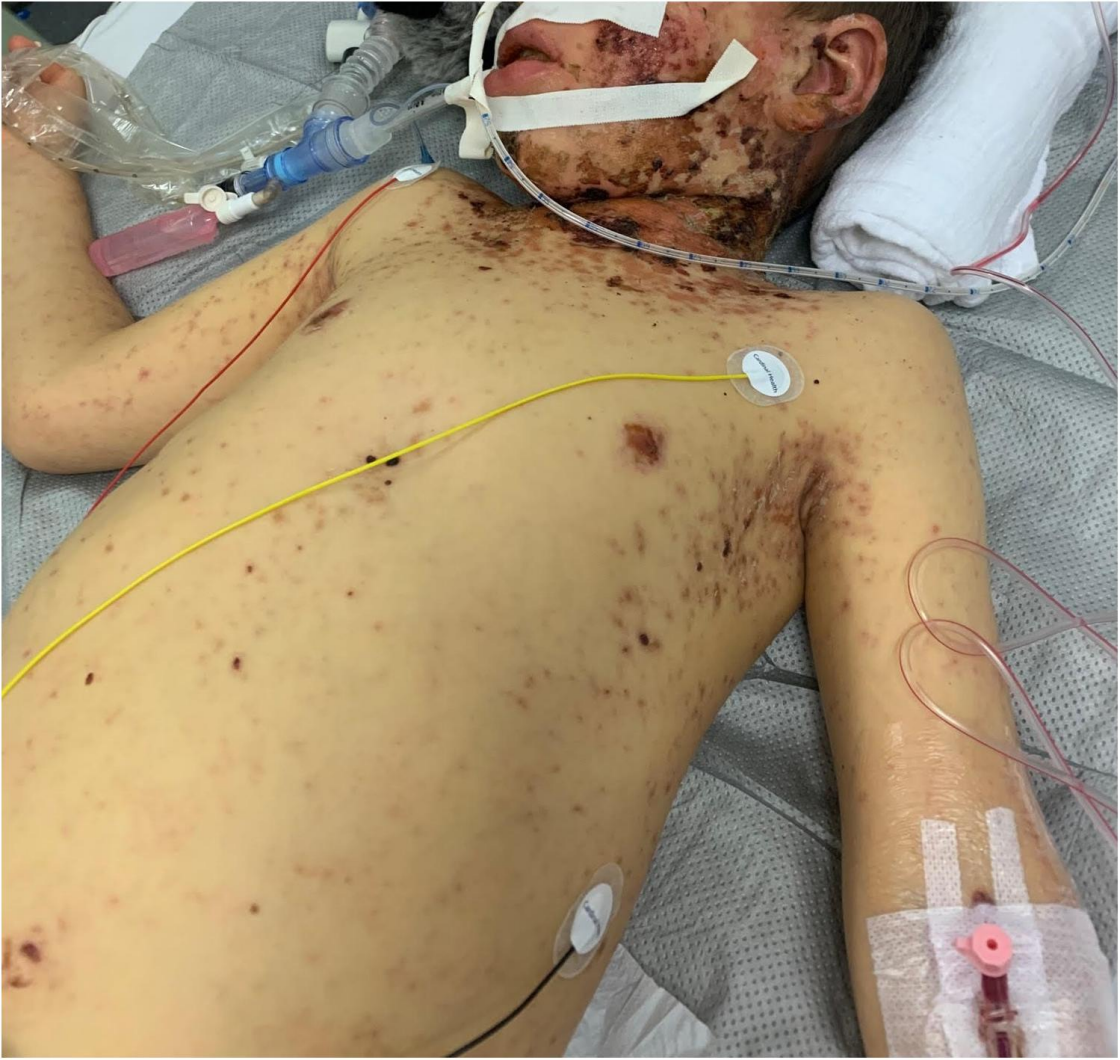
Six-year-old male patient on the seventh day after symptom onset, showing extensive epidermolysis characteristic of Stevens–Johnson syndrome. Published with written parental consent.

**Figure 2:**
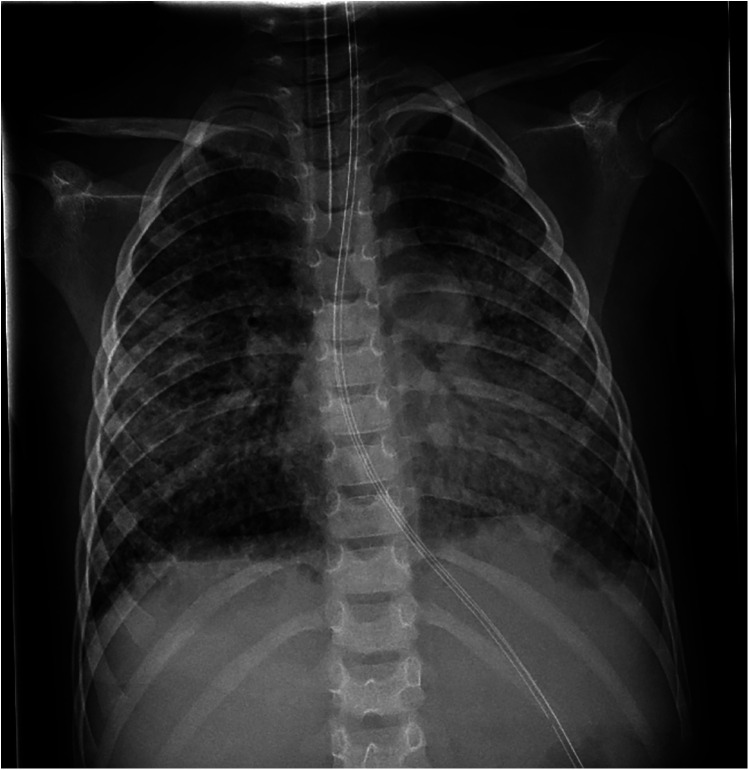
Chest x-ray on day 11 of illness, obtained at admission before ECMO cannulation, showing bilateral pulmonary opacifications consistent with severe ARDS.

On admission, he showed extensive cutaneous SJS involvement (see Figure 1) and severe respiratory insufficiency [pH 7.29, PaO_2_ 73 mmHg, PaCO_2_ 51 mmHg, FiO_2_ 1.0, positive end-expiratory pressure (PEEP) 15 mbar, peak inspiratory pressure (PIP) 50 mbar]. The calculated oxygenation index was 68, consistent with severe ARDS and meeting ECMO criteria. Chest x-ray demonstrated bilateral diffuse opacification (Figure 2). According to established severity assessment systems, the patient met high-risk thresholds by both the pediatric risk of mortality (PRISM) and pediatric logistic organ dysfunction (PELOD) criteria, reflecting multiorgan stress but preserved cardiac function. Broad microbiological and virological testing yielded negative results, and genetic as well as immunological work-up was unremarkable.

### ECMO support and adjunctive therapies

Rapid deterioration of the patient’s condition required initial VV-ECMO via a 19F bicaval double-lumen Avalon cannula, which was later supplemented by a femoral cannula due to recirculation. Lung-protective ventilation was applied. Adjunctive treatment included repeated intrapulmonary surfactant administration to improve alveolar recruitment as this therapy option has been reported to improve lung compliance and restore surfactant homeostasis (11); however, this treatment was discontinued after three applications due to the high cost and lack of result in our case. Corticosteroid therapy following the Meduri protocol was applied to modulate persistent inflammation and prevent fibroproliferative progression of ARDS (12, 13). Angiotensin-converting enzyme (ACE) inhibitor therapy was introduced to target pulmonary remodeling and fibrosis, as angiotensin-converting enzyme 2 (ACE2) is involved in the regulation of lung injury and repair pathways (14).

Oxygenators were exchanged regularly due to the risk of clot formation. Systemic anticoagulation was maintained with unfractionated heparin, targeting a partial thromboplastin time (PTT) of 50–60 s.

**Figure 3:**
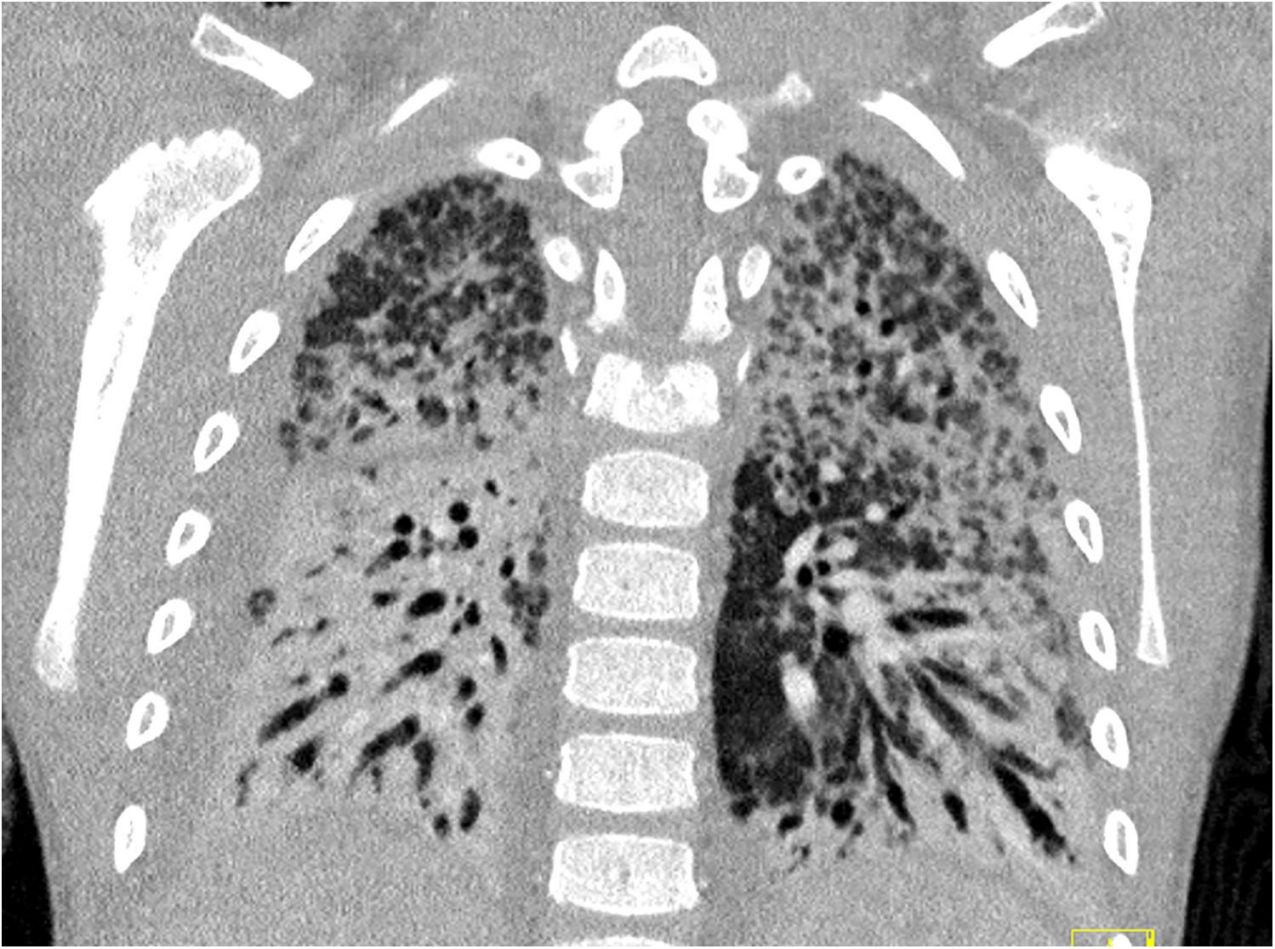
CT scan of the thorax during week 6 after symptom onset, demonstrating pulmonary fibrosis with only a few remaining areas of ventilation.

Despite therapy, the patient developed clinical and radiologic signs of pulmonary fibrosis (Figure 3). A tracheostomy was performed after 1 month. Lung compliance improved only after 8 weeks, allowing gradual ECMO weaning.

In addition to medical management, the patient received intensive multidisciplinary rehabilitation. Daily physiotherapy sessions were provided to preserve joint mobility, prevent contractures, and stimulate respiratory function. Once awake, the child also participated in occupational and speech therapy to support fine motor recovery, communication, and swallowing function.

### Weaning attempts

**Figure 4:**
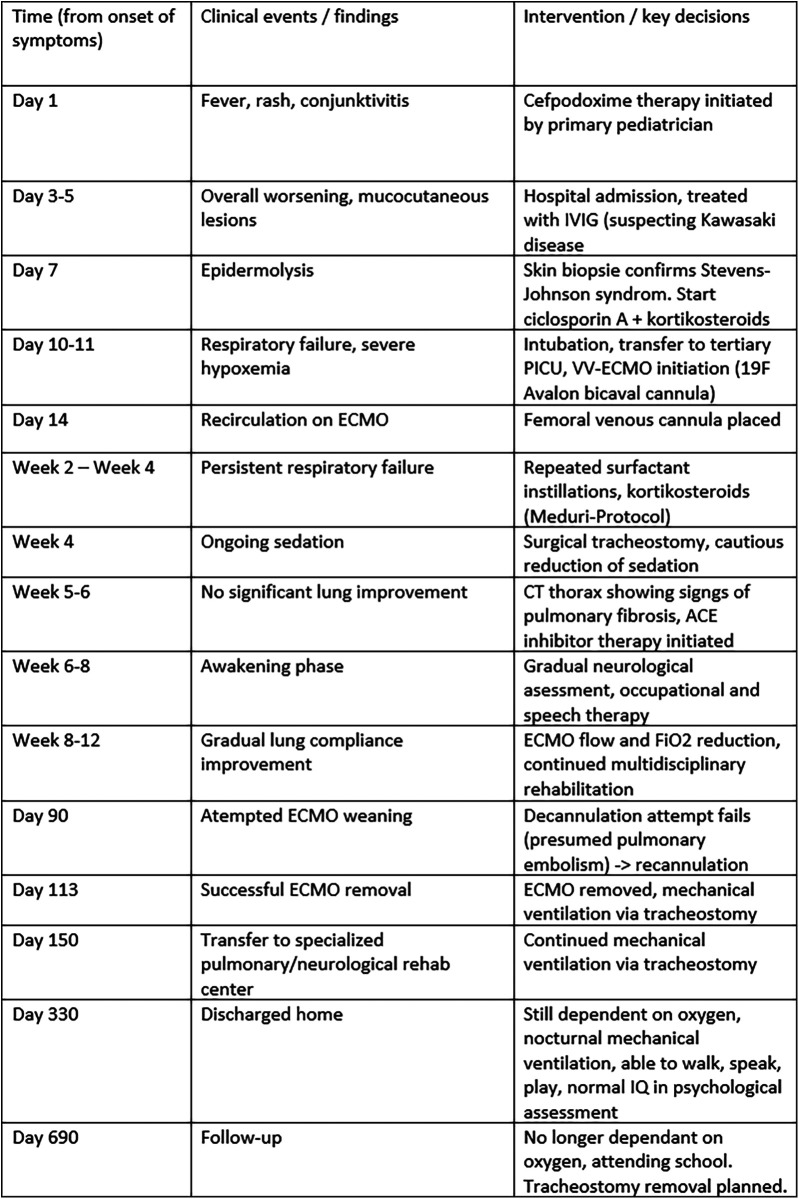
Clinical timeline summarizing major events from symptom onset to 18-month follow-up. The table displays the chronological sequence of key clinical events (left column), corresponding findings (middle column), and major interventions or management decisions (right column) throughout the patient's course. PICU, pediatric intensive care unit; IVIG, intravenous immunoglobulin; VV-ECMO, veno-venous extracorporeal membrane oxygenation; PEEP, positive end-expiratory pressure; PIP, peak inspiratory pressure; F, French size (outer diameter of the cannula).

After 90 days of ECMO, decannulation was attempted but failed due to severe tachypnea, likely related to pulmonary embolism. After this, recannulation was required. A second weaning attempt after three more weeks succeeded, and ECMO was finally removed after 113 days. The patient was discharged to rehabilitation on home ventilation and, 18 months later, no longer required respiratory support (see Figure 4).

### Hepatic complications

In addition to pulmonary failure, the patient presented with elevated transaminases and direct hyperbilirubinemia. After initiation of ECMO, liver enzyme levels and bilirubin continued to rise. Sonography revealed hepatomegaly with normal parenchymal echogenicity and preserved vascular flow. During the course of ECMO therapy, the patient required a total of 55 red blood cell transfusions due to hemolysis and circuit-related losses. Correspondingly, serum ferritin levels exceeded 12,000 ng/mL, and transferrin saturation was markedly elevated (185%), indicating significant secondary iron overload. A transjugular liver biopsy demonstrated mild portal hepatitis, cholestasis, and secondary hemochromatosis, but no fibrosis (Desmet stage 0). The iron overload was attributed to transfusion-related hemosiderosis and ongoing hemolysis, and chelation therapy with deferoxamine was initiated.

### Neurological complications

The patient was deeply sedated for the first 6 weeks of ECMO support, initially with benzodiazepines and opioids, and later transitioned to dexmedetomidine, ketamine, and inhalational sedation using isoflurane. Weekly sedation interruptions (“sedation holidays”) were attempted to assess neurological status. Awakening was markedly delayed, and the patient exhibited prolonged hypoactive delirium for approximately 3 weeks, requiring multimodal management and close supervision. Cranial CT scans showed only mild global brain atrophy without ischemic or hemorrhagic lesions.

After regaining consciousness, the patient demonstrated pronounced weakness and fine motor impairment consistent with critical illness polyneuropathy. An intensive, multidisciplinary rehabilitation program was initiated, including daily physiotherapy and several weekly sessions of occupational and speech therapy to support neuromotor recovery and communication skills. At discharge to the rehabilitation center, he was alert, communicative, and capable of basic motor tasks.

At the 18-month follow-up, formal neurocognitive assessment conducted by an external rehabilitation psychologist revealed age-appropriate attention span, memory, and language comprehension, with only mild residual fine motor limitations. The patient attends school and participates in age-typical activities. His IQ was tested within the normal range. Formal quality-of-life assessment using standardized pediatric tools (e.g., pediatric quality of life inventory (PedsQL), pediatric overall performance category (POPC), or pediatric cerebral performance category (PCPC) was not performed, as long-term follow-up is not conducted at our department.

### Ethical decision-making framework

The exceptionally long duration of ECMO therapy posed a challenge not only for the patient's parents but also for the multidisciplinary care team. Weekly meetings involving physicians, nursing staff, physiotherapists, and the psychosocial support team were held to review the patient's clinical progress, anticipate complications, and address emerging problems using a multimodal approach. In addition, structured weekly discussions were conducted with the parents by the ICU medical and nursing leadership together with the psychosocial team. Particular emphasis was placed on incorporating the wishes of the parents into ongoing care decisions. The criteria for therapy discontinuation were predefined, including the occurrence of intracranial hemorrhage or ischemic stroke. Conversion to veno-arterial ECMO in case of impaired cardiac function was consciously declined in view of the unfavorable risk–benefit ratio.

### Patient and family perspective

From the perspective of the patient’s family, the prolonged ECMO treatment was an emotionally and physically challenging journey. Regular meetings with the multidisciplinary team helped the family understand the therapeutic goals and risks, as well as limitations.

During the first months of treatment, the father took medical leave from work to remain at the hospital, while the mother—herself a trained nurse—actively supported the intensive care team and participated in her son's daily care. Their younger daughter was cared for mainly by grandparents during this time. Nevertheless, she visited her brother regularly in the hospital, even while he was still deeply sedated. Later, during the awake-ECMO phase, the siblings were able to spend time together, playing and interacting at the bedside, which helped the parents with their emotional recovery.

Apart from regular follow-up visits in the pulmonology and hepatology departments, family life has largely returned to normal. The family now looks forward to the closure of the tracheostomy, marking the final step in the patient’s recovery.

## Discussion

ECMO is a high-risk rescue therapy associated with frequent and potentially fatal complications (15). Survival rates for pediatric respiratory ECMO are approximately 49% (16), with VV-ECMO generally associated with lower mortality than veno-arterial extracorporeal membrane oxygenation (VA-ECMO) (17). While durations beyond 28 days have historically been linked to poor outcomes (8), isolated reports demonstrate that meaningful recovery is still possible after prolonged support. Our case underscores that extended ECMO runs may be justified when neurological status and other organ functions remain stable. Beyond the clinical and ethical considerations, prolonged ECMO support also poses substantial challenges in terms of healthcare resource utilization. Extended extracorporeal support requires continuous multidisciplinary staffing, repeated circuit and oxygenator exchanges, and long-term ICU occupancy—representing a considerable cost and logistical burden. In our healthcare setting, such prolonged support is fully covered by statutory health insurance, allowing medical decisions to be guided primarily by clinical and ethical reasoning rather than financial constraints. However, this situation may differ in other health systems, where reimbursement models or limited access to specialized ECMO centers can significantly influence decisions regarding continuation or withdrawal of support. In our case, the decision to proceed was justified by preserved neurological function, stable multiorgan status, and strong family engagement, indicating a meaningful chance for full recovery.

Neurological injury remains one of the major risks during ECMO due to systemic anticoagulation and circuit-related clotting (17, 18). In our patient, the absence of significant central nervous system (CNS) injury was a decisive factor in maintaining prolonged support. Multidisciplinary care, early tracheostomy, mobilization, and proactive management of complications were key contributors to survival and recovery.

Steroid therapy in pediatric ARDS remains controversial; however, we observed favorable outcomes with the use of methylprednisolone according to the Meduri protocol. This experience suggests that corticosteroids may warrant consideration in select cases of severe inflammatory ARDS.

## Conclusion

This case report describes one of the longest pediatric VV-ECMO runs with successful weaning. Despite pulmonary fibrosis and systemic complications, the patient recovered without severe neurological sequelae. Prolonged ECMO should remain an individualized decision, but our experience supports continuation in selected cases, provided neurological status remains intact and no contraindications arise.

## Data Availability

The original contributions presented in the study are included in the article/Supplementary Material, further inquiries can be directed to the corresponding author.
